# Effects of a Proprietary Freeze-Dried Water Extract of *Eurycoma longifolia* (Physta) and *Polygonum minus* on Sexual Performance and Well-Being in Men: A Randomized, Double-Blind, Placebo-Controlled Study

**DOI:** 10.1155/2014/179529

**Published:** 2014-01-12

**Authors:** Jay K. Udani, Annie A. George, Mufiza Musthapa, Michael N. Pakdaman, Azreena Abas

**Affiliations:** ^1^Medicus Research LLC, Northridge, CA 91325, USA; ^2^Northridge Hospital Integrative Medicine Program, Northridge, CA 91325, USA; ^3^Biotropics Malaysia Berhad, Level 52, Menara TM, Jalan Pantai Baharu, 50672 Kuala Lumpur, Malaysia

## Abstract

*Background*. Physta is a proprietary product containing a freeze-dried water extract of *Eurycoma longifolia* (tongkat ali), which is traditionally used as an energy enhancer and aphrodisiac. We aim to evaluate a 300 mg combination of Physta and *Polygonum minus*, an antioxidant, with regard to sexual performance and well-being in men. *Methods*. Men that aged 40–65 years were screened for this 12-week randomized, double-blind, placebo-controlled, parallel-group study. Outcome measures included validated questionnaires that aimed to evaluate erectile function, satisfaction with intervention, sexual intercourse performance, erectile hardness, mood, and overall quality of life. *Results*. 12 subjects in the active group and 14 in the placebo group completed the study. Significant improvements were noted in scores for the Sexual Intercourse Attempt diary, Erection Hardness Scale, Sexual Health Inventory of Men, and Aging Male Symptom scale (*P* < 0.05 for all). Three adverse events were reported in the active group and four in the placebo group, none of which were attributed to study product. Laboratory evaluations, including liver and kidney function testing, showed no clinically significant abnormality. *Conclusion*. Supplementation for twelve weeks with *Polygonum minus* and the proprietary *Eurycoma longifolia* extract, Physta, was well tolerated and more effective than placebo in enhancing sexual performance in healthy volunteers.

## 1. Introduction

Erectile dysfunction (ED) occurs as a result of a lack of blood flow to the penis, impairing the ability to achieve or maintain an erection suitable for sexual intercourse [1]. This condition affects approximately 30 million men in the United States and approximately 52% of men aged 40–70 years worldwide [1]. While the incidence of erectile dysfunction increases with age, men at all ages are at risk. Risk factors for ED include heart disease, hypertension, hyperlipidemia, diabetes, prostate disease, depression, stress, anxiety, smoking, and prescription/recreational drug use.

Testosterone is a steroid hormone that plays a key role in development and maintenance of the male reproductive system. As men age, the endogenous production of testosterone by the Leydig cells tends to decrease at a rate of 1.6% per year [2]. Decreased levels of testosterone, including levels that are still within the normal range, have been linked to erectile dysfunction, particularly in older men [2–4].

The most popular prescription medications aimed at treating ED today are classified as cGMP-specific phosphodiesterase type 5 (PDE5) inhibitors, which act by regulating blood flow to the penis. Examples of such medications include sildenafil (Viagra), tadalafil (Cialis), and vardenafil (Levitra) [5–7]. However, side effects from these medications as well as their interactions with vasodilator therapy have left many men to turn to more natural options for management of their sexual health [8–10].

*Eurycoma longifolia *(Physta) is a tree native to the jungles of Malaysia, Thailand, and Indonesia and is believed to enhance sexual performance. The root of *Eurycoma longifolia* has been promoted as a tonic, energy enhancer, and aphrodisiac [11–13]. An unpublished *in vitro* study demonstrated a fourfold increase in testosterone levels among human testicular cells when exposed to *Eurycoma longifolia*. *Eurycoma longifolia* has been found to facilitate conversion of pregnenolone to progesterone, cortisol, 5-dehydroepiandrosterone (DHEA), and testosterone in rabbit corpus cavernosum tissues [14]. An animal study has shown that consumption of *Eurycoma longifolia* in sexually sluggish and impotent male rats can lead to increased serum testosterone levels, reduced ejaculation latencies, and an increased likelihood of mounting and ejaculating [15]. Toxicity evaluation on *Eurycoma longifolia* has been performed in humans and the acceptable daily intake was determined to be 1.2 g per day for an adult male [16].

*Polygonum minus* is an aromatic plant originating from southeast Asia, belonging to the family Polygonaceae. It has flavonoid properties and is widely used as a food additive [17]. It has been described to have antioxidant activity, antimicrobial activity, and antiulcer activity [18]. There are no known interactions between *Eurycoma longifolia* and *Polygonum minus*.

Validated measures of male sexual health aim to measure subjective satisfaction, penile hardness, frequency and quality of sexual intercourse attempts, and overall physical activity. The erectile dysfunction inventory for treatment satisfaction (EDITS) questionnaire is a 5-point scale ranging from 0 to 4 [19]. This scale is used to evaluate change over time in response to treatment. The sexual intercourse attempt (SIA) diary consists of 10 yes/no questions evaluating erectile function in healthy subjects. The EHS is a four-point scale used to evaluate changes in hardness by rating hardness during each sexual intercourse attempt on a scale of 1 to 4. A higher score indicates increased satisfaction with erectile hardness [7]. The sexual health inventory for men (SHIM) questionnaire is a six-point scale aimed at identifying the degree of erectile function, with a score of 21 or below indicating erectile dysfunction [6]. The aging male symptom score (AMS) is a six-point Likert scale ranging from 0 to 5 measuring satisfaction with overall physical function. A higher score indicates worse function [20]. The index of erectile function (IIEF-5) is a six-point scale that aims to evaluate the effect of erection problems on an individual's sex life over the previous four weeks [21]. The self-esteem and relationship (SEAR) questionnaire is a patient-reported measure of psychosocial variables that uses a six-point Likert scale ranging from 0 to 5 to evaluate confidence, self-esteem, and the quality of sexual relationships [5]. The beck depression inventory (BDI) and beck anxiety inventory (BAI) score individual items on a four-point scale (0–3) to subjectively evaluate the degrees of depression and anxiety, respectively [22, 23].

This randomized, double-blind, and placebo-controlled pilot study will provide valuable data with regard to the proposed effects of the *Eurycoma longifolia* containing proprietary product, Physta, in improving sexual performance, quality of life, and well-being.

## 2. Methods

### 2.1. Investigational Product

The investigational product for this study was a combination of *Eurycoma longifolia* and *Polygonum minus *extracts. *Eurycoma longifolia* was prepared as Physta, a proprietary freeze-dried water extract of *Eurycoma longifolia* root with a drug-extract ratio (DER) of 1 : 25. *Polygonum minus *leaves were prepared as a water extract with a DER of 1 : 10. The study product tablets were produced under continuous quality of good manufacturing process requirements by Biotropics Malaysia (Berhad, Kuala Lumpur, Malaysia). The placebo was prepared as a sensory-identical tablet composed of *α*-lactose-monohydrate, microcrystalline cellulose, and magnesium stearate. The active product contained 200 mg of *Eurycoma longifolia *and 100 mg of *Polygonum minus*. Dosage instructions for each product were to take one tablet per day.

### 2.2. Subjects

We aimed to enroll healthy male participants between the ages of 40 and 65 years. Subjects were recruited from the community by methods including advertisements and recruitment databases. Phone screening was performed prior to scheduling an inclinic screening visit. Participants were required to be in a stable heterosexual relationship for at least six months. Both partners had to agree to attempt intercourse at least once a week, on average, during the study. Exclusion criteria are outlined in Table 1.

### 2.3. Study Design

We aimed to enroll 30 men in this randomized, double-blind, and placebo-controlled parallel-design study. Institutional Review Board (IRB) approval was obtained (Copernicus Group IRB, Cary, NC, USA) prior to the initiation of any study-related activities. Simple randomization was prepared using a computer program based on the atmospheric noise method and sequential assignment was used to determine group allocation [24]. Subjects, clinical staff, data management staff, and statistical analysis staff were unaware of the study group. The study was conducted at the Staywell Research clinical research site located in Northridge, California.

An inclinic screening visit was performed 2 weeks prior to the baseline visit. At screening, inclusion and exclusion criteria were reviewed, vital signs were measured, and a generalized history and physical examination were performed. Labs performed at screening included safety labs (CBC, CMP, and urinary analysis) as well as serum total and free testosterone. Questionnaires were administered to subjects to confirm eligibility. These included the BPH Symptom Score, the International Index of Erectile Function (IIEF), the Sexual Health Inventory for Men (SHIM) Questionnaire, the Beck Depression Index, the Beck Anxiety Index, and the Alcohol/Compliance Questionnaire.

Subjects who passed screening were randomized on visit 2 (week 0). Baseline endpoint data was collected and product was dispensed. Subjects returned for two more visits at week 6 and week 12, where completed diaries and any remaining study product were returned, labs were drawn, and a series of scales and questionnaires were administered. Sexual intercourse diaries were completed after each sexual intercourse attempt. Safety labs were repeated at the conclusion of the study at week 12.

Throughout the course of the study, subjects were instructed not to alter their current diet and exercise habits. The Bodymedia Fit System Armband was used to collect activity data. A breakdown of all study evaluations and procedures is demonstrated in Table 2.

### 2.4. Endpoints

The primary objective of this study was to compare the proprietary tongkat ali/*Polygonum minus* combination to placebo with regard to sexual performance. Measured endpoints for this objective included the following series of subjective surveys: the EDITS questionnaire, the SIA diary, the EHS scale, the SIA logs, the EHS scale, the SHIM questionnaire, the AMS score, and the IIEF-5.

For the SIA diary, “sexual activity” was defined as partial penile entry into the partner's vagina, while “sexual intercourse” was defined as penetration of the entire penile shaft into the partner's vagina. “Ejaculation” was defined as the ejection of semen from the penis. “Foreplay” consists of kissing, hugging, touching, intimacy, masturbation, and/or oral sex.

The secondary objective was to compare the proprietary tongkat ali/*Polygonum minus* combination to placebo on quality of life endpoints. This quality of life endpoints included the SEAR Questionnaire, the Beck Depression Index, and the Beck Anxiety Index. A summary of all the scales and questionnaires used in this study is summarized in Table 3.

The tertiary objective of this study was to compare a proprietary tongkat ali/*Polygonum minus* combination to placebo on serum testosterone levels (total and free). The normal ranges for total testosterone are 350–1.200 ng/dL and 5.4–12.3 ng/dL for free testosterone. Analysis involved immunoassay for total testosterone and radioimmunoassay (RIA) for the free testosterone. Laboratory testing was performed by Primex Laboratories (Van Nuys, CA).

Additional endpoints included height, weight, waist circumference, hip circumference, body fat percentage, and armband data. Safety endpoints included complete blood count, comprehensive metabolic panel, urinary analysis, at week 6 and week 12, and adverse event monitoring.

### 2.5. Statistics

Completers analysis was performed, including only those who completed all study visits toward the final data set. Paired sample *t*-tests were used within subject means comparisons and independent sample *t*-tests between group comparisons (placebo versus active group). Statistical analyses were performed using SPSS Base System ver. 18 (IBM SPSS Inc., Chicago, IL, USA). Significance was indicated at *P* < 0.05.

## 3. Results

**Figure 1:**
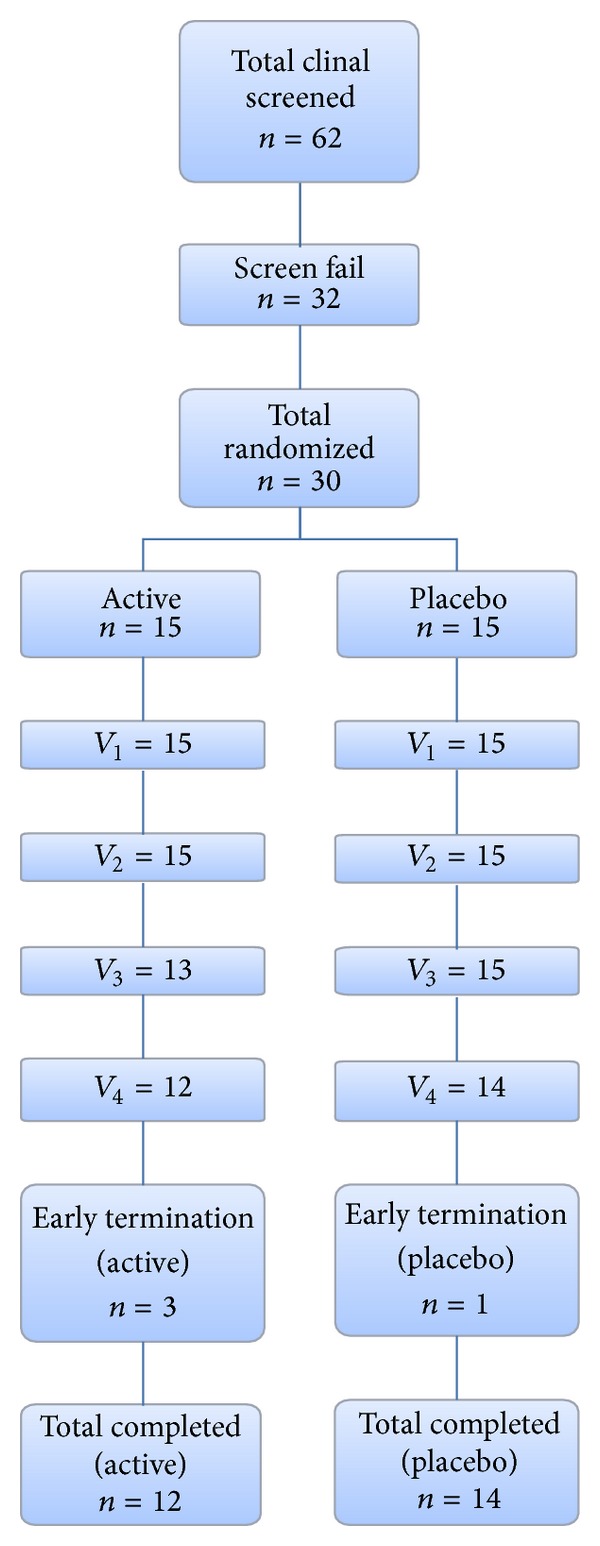
Attrition chart.

62 subjects were screened and 30 were randomized to either placebo (*n* = 15) or product (*n* = 15). Four subjects terminated the study early due to relocation, uncontrolled bowel movement, loss to follow-up, or voluntary subject withdrawal. The complete attrition chart is demonstrated in Figure 1.

Satisfaction with the study product, as measured by the EDITS Questionnaire, showed no significant difference between the active group and placebo group at six or 12 weeks (*P* > 0.05). However, within-group analysis of the active product group demonstrated a significant increase in satisfaction when comparing six to 12 weeks (*P* = 0.027). Analysis of the placebo group showed no significant change (Table 4).

Analysis of the SIA questionnaire revealed a significant change from baseline to 12 weeks in the active group as compared to the placebo group in 7 of the 11 scales. At 12 weeks, a significant improvement was noted in the ability to insert the entire shaft into the partner's vagina, the overall satisfaction with the sexual experience, and the overall score for the erection during the SIA. Subjects also noted a significant increase in the elapsed time from erection perceived hard enough for penetration to withdrawal from partners vagina (7.47 minutes at baseline to 19.56 minutes at 12 weeks, *P* < 0.05). No significant changes were noted in the placebo group with respect to these variables (Table 5).

Only two questions on the SIA questionnaire were noted to have a significant improvement among the placebo group from baseline to 12 weeks. These included the frequency of ejaculating while still inside the partner and the overall satisfaction with the hardness of erection. These responses also showed significant improvement in the active group (Table 5).

The mean baseline scores on the EHS were 2.54 for the active group and 2.14 for the placebo group. Among the active group, a statistically significant improvement in EHS score was noted at 6 and at 12 weeks when compared to baseline (*P* < 0.05). No significant changes were noted in the placebo group. The SHIM score demonstrated a significant improvement from baseline to week 12 in the active group only (*P* < 0.005). The 12-week SHIM score of 19.85 was also significantly higher than the placebo group, which was 14.29 (*P* < 0.005). Analysis of the AMS scale at 12 weeks showed significant improvement from baseline among the active group only. This 12-week score in the active group (20.85) was also significantly improved from the placebo group (26.0, *P* = 0.037).

Analysis of the IIEF-5 responses showed no significant difference between the groups or when compared to baseline.

With respect to the SEAR questionnaire, overall relationship satisfaction decreased in both groups and was significantly lower in the active group at six and 12 weeks (*P* = 0.012 at 12 weeks). The decrease from baseline was significant at both six and 12 weeks in the active group and was not significant in the placebo group. Sexual relationship satisfaction was significantly lower in the active group compared to the placebo group at baseline and at 12 weeks (*P* < 0.0001). The reduction from baseline was significant for the placebo group at six weeks only. Self-esteem scores showed no significant change in either group.

Analysis of the BDI and BAI showed no significant difference between active and placebo groups and no significant change from baseline over time.

Total testosterone levels increased from a baseline value of 359.2 ng/dL in the active group and 308.5 ng/dL in the placebo group to 396.4 ng/dL and 32.7 ng/dL at 12 weeks, respectively (*P* < 0.005 for active and *P* < 0.05 for placebo). Free testosterone levels also demonstrated a statistically significant decline in both groups (*P* < 0.05) (Table 6).

Armband data did not show any significant differences between groups regarding energy expenditure or physical duration. There were no significant differences in vital signs between groups or compared with baseline.

There were no significant changes in weight from baseline in either group and no significant difference between groups with regard to weight measurement. There were no significant changes in waist measurement from baseline in either group; however, the waist measurements in the active group were significantly lower than those of the placebo group at week 6 (*P* = 0.032) and week 12 (*P* = 0.016). The hip measurements did not demonstrate any change from baseline in either active or placebo group; however, hip measurements in the active group were significantly lower than those of the placebo group at week 12 (*P* = 0.015).

With regard to the safety profile, we noted no clinically significant changes in any of the laboratory parameters throughout the study. Our study shows no significant changes from baseline or against placebo in relevant liver and kidney lab values, including albumin, aspartate aminotransferase (AST), alanine aminotransferase (ALT), alkaline phosphatase, bilirubin, blood urea nitrogen (BUN), creatinine, or calculated glomerular filtration rate (GFR) (Table 7). No adverse events were attributed to the test product. No serious adverse events were reported.

## 4. Discussion

*Eurycoma longifolia* has been historically used as an aphrodisiac in Asian countries for many years. In this study, a proprietary freeze-dried water extract of *Eurycoma longifolia, *Physta, was investigated for its effect on sexual performance, satisfaction, and well-being in subjects who desired improved sexual performance. Our study demonstrates significantly improved subjective libido scores as well as improved sexual performance when compared to placebo. The results also suggested a potential benefit to anthropometric measures including waist and hip measurements.

A recent study by Ismail et al. enrolled 109 men between 30 and 55 years of age in a randomized, double-blind, and placebo-controlled study on *Eurycoma longifolia* and sexual well-being. They found a significant improvement in SF-36 scores, a scale evaluating overall physical functioning. They also noted higher scores in overall erectile function per the SIA as well as improved sexual libido. Their finding of decreased fat mass in subjects with BMI > 25 kg/m^2^ corroborates with our findings of decreased waist and hip circumference, suggesting a role of *Eurycoma longifolia *in weight loss [25]. A recent open-label study on subjects with late-onset hypogonadism used a water soluble extract of *Eurycoma longifolia* and demonstrated improved AMS score and increased serum testosterone levels [26].

The potential mechanism of action for *Eurycoma longifolia* as an aphrodisiac is not yet clear. *Eurycoma longifolia* is rich in compounds such as eurycomaoside, eurycolactone, eurycomalactone, eurycomanone, and pasakbumin-B, which may play a role in its aphrodisiac properties [27]. *Eurycoma longifolia* is often referred to as an adaptogen, an agent that may rejuvenate the body through restoration [14, 28–30].

Several animal studies have shown that *Eurycoma longifolia* intake resulted in increased sexual interest and arousal [15, 31–37]. Among *Andrographis paniculata* induced infertile rats, *Eurycoma longifolia* was found to increase serum testosterone as well as sperm count, motility, and morphology [15, 38]. Our study revealed an increase in total testosterone by 10.36% in the treatment group and 4.28% in the placebo group. The finding of increased levels of total testosterone and decreased levels of free testosterone poses many questions regarding testosterone metabolism. *Eurycoma longifolia* may play a role in increasing production of sex-hormone binding globulin. Alternatively, supplementation with *Eurycoma longifolia* may lead to increased metabolism and breakdown of free testosterone. Studies supporting this hypothesis have shown that abstinence appears to increase testosterone levels [39, 40]. As a higher standard deviation was observed in the treatment group after 12 weeks of supplementation, considering the adaptogenic nature of the herb, it may be postulated that the herb was most effective in subjects with lower baseline levels of testosterone [26, 29, 30].

In our study, satisfaction scores did not improve in either group. This could be related to increased anxiety related to the testing atmosphere or due to unrealistic expectations of the study product. Thus while the SIA questionnaire demonstrated significant improvements in sexual performance, if these improvements did not meet the subject's expectations, then the subject may note lower satisfaction scores.

With regard to product toxicity, animal studies on toxicity showed no toxicity effects of *Eurycoma longifolia* on Wistar rats in acute (2000 mg/kg), subacute (250, 500, and 1000 mg/kg over 28 days), or chronic (250, 500, and 1000 mg/kg over 90 days) dosing periods. No mortality or changes in physiological activities were noted [41]. Over our study period of 12 weeks (84 days), no adverse events occurred that were attributable to study product. Vital sign measurements and routine blood work, including complete blood count (CBC) and complete metabolic profile (CMP), also showed no significant change. Additionally, this is the first clinical trial to evaluate the safety of the *Polygonum minus* extract, and from our study we find that *Polygonum minus* is safe to consume in the study population represented here. No adverse interactions were noted from the combination of *Eurycoma longifolia* and *Polygonum minus*.

A common safety concern among those concerns considering consumption of a natural product is liver and kidney toxicity. Our study shows no significant changes from baseline or against placebo in relevant liver and kidney lab values, including albumin, AST, ALT, alkaline phosphatase, bilirubin, BUN, creatinine, and calculated GFR (Table 7).

A major limitation of this study involves the nature of the endpoints being measured. Requiring data collection with regard to sexual performance compromises the goal of inducing a physiologic state that “by its inner nature, is deeply linked with issues of intimacy, privacy, and sexual arousal” [42]. Requiring individuals to think about and record their degree of hardness interferes with the intimate environment in which individuals commonly achieve and maintain erections. Testing in this manner introduces confounders related to anxiety and unfamiliarity [43]. Additionally, participants in this study represent a nondiseased (healthy) population. These individuals are not particularly familiar with a clinical environment and may be uncomfortable sharing intimate details regarding their sex life. The act of discussing and filling out surveys regarding desire, performance, and satisfaction may lead to laboratory-induced inhibition and decreased sexual drive [42, 44].

In conclusion, Physta significantly improved sexual performance across several clinically important parameters after 12 weeks when compared to placebo. In addition, the product was well tolerated, with excellent safety profiles (identical to placebo) at the administered dose of 300 mg daily for 12 weeks. However, the study product was not able to improve satisfaction with sexual performance. The results of this study suggest that this product may be of interest to generally healthy middle-aged men who desire improved sexual performance and health. Further research is still needed in order to confirm the mechanism of action in human males. Also, there is a need for further research in younger and older men, in men with cardiovascular disease and diabetes, and in individuals using medications to treat ED and other disorders.

## Figures and Tables

**Table 1:** Inclusion criteria, exclusion criteria, and study controls.

Inclusion criteria	
Male	
Age between 40 and 65 years	
In a stable heterosexual relationship for at least 6 months	
Testosterone levels ≤450 ng/dL	
Index of erectile dysfunction scores of 17–25	
Exclusion criteria	
History of prostate cancer	
Elevated prostate-specific antigen (PSA)	
Benign prostate hypertrophy (BPH) scores ≥40	
Penile anatomical abnormalities	
Premature ejaculation	
Cardiovascular disease	
Resting hypotension (resting systolic blood pressure 90 mmHg)	
Resting hypertension (resting systolic blood pressure >170 mmHg or diastolic pressure >110 mmHg)	
Primary hypoactive sexual desire	
Abnormal prostate exam during the screening visit	
Study controls	
Both partners had to agree to attempt intercourse at least once per week on average during the study	

**Table 2:** Visit details.

	V1	V1.5	V2	V3	V4
	Screening visit		Baseline Visit		
	week 2		Week 0	Week 6	Week 12
Protocol activity					
Informed consent	x	Randomization assignment and paperwork	—	—	—
Inclusion/exclusion	x		—	—	—
Medical/medication history	x		—	—	—
Intercurrent medical issues/AE review	—		x	x	x
Review concomitant therapies	x		x	x	x
Physical examination (including prostate exam)	x		—	—	x
Randomization	—		x	—	—
Vital signs	x		x	x	x
Anthropomorphic measures	x		—	x	x
BIA	—		x	x	x
Dispense bodymedia armband	—		x	x	x
Dispense subject diaries	x		x	x	—
Collect subject diaries	—		x	x	x
Dispense product	—		x	x	—
Pill count/compliance assessment	—		—	x	x
Administer scales and questionnaires					
Benign prostate hyperplasia (BPH) symptom score	x		—	—	—
Index of erectile function (IIEF-5)	x		x	x	x
Erectile dysfunction inventory for treatment satisfaction (EDITS) questionnaire	—		—	x	x
Sexual health inventory questionnaire	x		—	—	—
Aging males symptom score (AMS)	—		x	x	x
Self-esteem and relationship questionnaire (SEAR)	—		x	x	x
Beck depression index	x		x	x	x
Beck anxiety index	x		x	x	x
Alcohol/compliance questionnaire	x		—	—	—
Laboratory					
Total + free testosterone	x		—	x	x
CBC	x		—	—	x
CMP	x		—	—	x
UA	x		—	—	x

**Table 3:** Scales and ranges.

Scales and scoring	Range	Explanation
EDITS	0–4	Satisfaction with current treatment or intervention
SIA	Yes/No	Subjective rating of erectile function
EHS	1–4	Rate current level of hardness during intercourse
SHIM	0–21	Used in assessment of erectile dysfunction
AMS	0–5	Satisfaction with overall physical function
IIEF-5	0–5	Erection problems in sex life over 4-week interval
SEAR	0–5	Questionnaire on psychosocial variables
BDI	0–3	Evaluates degree of depression
BAI	0–3	Evaluates degree of anxiety

EDITS: Erectile Dysfunction Inventory for Treatment Satisfaction.

SIA: Sexual Intercourse Attempts.

EHS: Erection Hardness Scale.

SHIM: Sexual Health Inventory Questionnaire for Men.

AMS: Aging Male Symptom Score.

IIEF-5: Index of Erectile Function.

BDI: Beck Depression Inventory.

BAI: Beck Anxiety Inventory.

**Table 4:** Effect of treatment on various sexual dysfunction endpoints.

	Baseline (mean ± SE)		6 weeks (mean ± SE)		12 weeks (mean ± SE)	
	Active	Placebo	Active	Placebo	Active	Placebo
Erectile Dysfunction Inventory of Treatment Satisfaction (EDITS)	Not possible to score	Not possible to score	52.56 ± 6.80	68.59 ± 8.03	74.68 ± 8.98	78.53 ± 9.89
Erection Hardness Scale (EHS)	2.54 ± 0.22	2.14 ± 0.23	2.95 ± 0.22^d^	2.68 ± 0.15	3.54 ± 0.11^b,c^	2.87 ± 0.27
Sexual Health Inventory for Men (SHIM)	15.77 ± 1.32	12.36 ± 1.45	16.92 ± 1.46	14.21 ± 1.56	19.85 ± 1.21^a,c^	14.29 ± 1.81
Aging Males Symptom (AMS) scale	25.85 ± 2.02	29.43 ± 2.25	23.31 ± 1.54^d^	24.71 ± 1.79^c^	20.85 ± 1.10^b,d^	26.00 ± 2.78

^a^ *P* < 0.005 versus placebo at this time point.

^b^ *P* < 0.05 versus placebo at this time point.

^c^ *P* < 0.005 versus baseline in the treatment group.

^d^ *P* < 0.05 versus baseline in the treatment group.

**Table 5:** Effect of treatment on sexual intercourse assessment (SIA).

SIA questions	Baseline (mean ± SE)		6 weeks (mean ± SE)		12 weeks (mean ± SE)	
	Active	Placebo	Active	Placebo	Active	Placebo
Was this the first attempt at sexual intercourse on this day?	1.00 ± 0.00^a^	0.97 ± 0.026	0.98 ± 0.023	0.98 ± 0.017	0.96 ± 0.038	0.99 ± 0.009
Was foreplay or sexual activity initiated with the goal of sexual intercourse?	1.00 ± 0.00^a^	0.95 ± 0.04	0.96 ± 0.03	0.95 ± 0.03	0.95 ± 0.05	0.98 ± 0.02
Did foreplay precede the attempted intercourse?	1.00 ± 0.00^a^	0.97 ± 0.02	0.97 ± 0.03	0.95 ± 0.03	0.93 ± 0.05^a^	0.98 ± 0.02
Were you able to achieve at least some erection?	1.00 ± 0.00^a^	0.95 ± 0.05	0.98 ± 0.02	0.95 ± 0.02	1.00 ± 0.00^b^	0.97 ± 0.02
Were you able to insert your entire penile shaft into your partner's vagina?	0.71 ± 0.11	0.42 ± 0.13	0.84 ± 0.07	0.64 ± 0.09	0.96 ± 0.02^b,c^	0.62 ± 0.13
Did your erection last long enough for you to have successful intercourse?	0.44 ± 0.12	0.35 ± 0.12	0.67 ± 0.09^c^	0.54 ± 0.10	0.89 ± 0.04^b^	0.60 ± 0.13
Elapsed time from erection perceived hard enough for penetration to withdrawal from your partner's vagina (in minutes)?	7.47 ± 2.06	7.06 ± 1.82	11.81 ± 2.65	7.27 ± 1.67	19.56 ± 3.93^c^	12.28 ± 3.17
Did you ejaculate while still in your partner?	0.29 ± 0.11	0.33 ± 0.12	0.52 ± 0.10^c^	0.53 ± 0.12	0.62 ± 0.11^c^	0.65 ± 0.12^c^
Overall, were you satisfied with the hardness of your erection?	0.28 ± 0.11^b^	0.06 ± 0.03	0.55 ± 0.10^c^	0.24 ± 0.09	0.70 ± 0.09^a,d^	0.35 ± 0.13^c^
Overall, were you satisfied with this sexual experience?	0.33 ± 0.10	0.33 ± 0.13	0.61 ± 0.10^c^	0.44 ± 0.11	0.87 ± 0.06^b,e^	0.52 ± 0.14
Please rate the range of your erection during this sexual intercourse attempt.	2.54 ± 0.22	2.14 ± 0.23	2.95 ± 0.22^c^	2.68 ± 0.15	3.54 ± 0.11^a,d^	2.87 ± 0.27

^a^ *P* < 0.05 versus placebo at this time point.

^b^ *P* < 0.0005 versus placebo at this time point.

^c^ *P* < 0.05 versus baseline in the treatment group.

^d^ *P* < 0.005 versus baseline in the treatment group.

^e^ *P* < 0.0005 versus baseline in the treatment group.

**Table 6:** Effect of treatment on testosterone levels.

	Baseline (mean ± SE)		6 weeks (mean ± SE)		12 weeks (mean ± SE)	
	Active	Placebo	Active	Placebo	Active	Placebo
Total testosterone	359.23 ± 27.09	308.47 ± 23.70	396.54 ± 36.41^a^	334.33 ± 27.86^a^	396.46 ± 47.26^a^	321.67 ± 27.51^b^
Free testosterone	10.73 ± 1.12	10.43 ± 0.72	10.14 ± 1.08^b^	8.34 ± 0.57	8.55 ± 1.07^b^	7.33 ± 0.82^b^

^a^ *P* ≤ 0.005 versus baseline in the treatment group.

^b^ *P* ≤ 0.05 versus baseline in the treatment group.

**Table 7:** Safety lab values.

		Baseline	12 weeks	Difference	Significance
Albumin	Active	4.4 ± 0.2	4.2 ± 0.30	−0.20	*P* = 0.351
	Placebo	4.29 ± 0.018	4.3 ± 0.05	0.01	
AST	Active	22.33 ± 5.55	20.92 ± 4.41	−1.40	*P* = 0.406
	Placebo	22.64 ± 8.61	21.71 ± 6.10	−0.90	
ALT	Active	21.87 ± 9.26	22.31 ± 9.97	0.44	*P* = 0.243
	Placebo	22.29 ± 12.57	23.71 ± 8.71	1.42	
Alkaline phosphatase	Active	77.73 ± 19.19	68.85 ± 19.0	−8.90	*P* = 0.573
	Placebo	75.00 ± 15.85	70.43 ± 19.84	−4.60	
Total bilirubin	Active	0.78 ± 0.44	0.78 ± 0.63	0.00	*P* = 0.271
	Placebo	0.73 ± 0.18	0.66 ± 0.24	−0.10	
BUN	Active	16.33 ± 4.82	17.23 ± 3.63	0.90	*P* = 0.668
	Placebo	16.86 ± 4.74	17.57 ± 5.37	0.71	
Creatinine	Active	1.08 ± 0.13	1.08 ± 0.13	0.00	*P* = 0.267
	Placebo	1.09 ± 0.16	1.05 ± 0.23	−0.00	
Estimated GFR	Active	77.0 ± 11.7	78.08 ± 10.3	1.08	*P* = 0.296
	Placebo	77.21 ± 15.2	82.21 ± 20.2	5.00	
